# Uncommon Presentation of a Common Tachycardia

**Published:** 2010-09-05

**Authors:** Raja Selvaraj, Palamalai Arunprasath, Balakrishnan Karthikeyan, Geofi George, Jayaraman Balachander

**Affiliations:** Department of Cardiology, Jawaharlal Institute of Postgraduate Medical Education and Research, Puducherry, India

**Keywords:** atrioventricular nodal reentrant tachycardia, incessant tachycardia

## Abstract

We describe a patient with an implanted pacemaker for impaired AV conduction who presented with  an incessant tachycardia. EP study showed that the tachycardia was atrioventricular nodal reentrant tachycardia (AVNRT) with repeated spontaneous initiation because of poor or absent antegrade fast pathway conduction. Slow pathway ablation was successful in terminating the tachycardia and making it non-inducible.

## Case Report

During a follow-up visit in the pacemaker clinic, a 45-year-old male complained of persistent palpitations for the past 2 months. He had undergone implantation of a permanent single chamber pacemaker with a ventricular lead 18 years back for trifascicular block with intermittent complete heart block and had subsequently undergone pulse generator replacement twice. His heart rate in the clinic was 130 bpm. Echocardiogram showed normal LV function without any abnormalities. He was not on any drugs. Electrocardiogram showed a broad complex tachycardia with a right bundle branch block (RBBB) morphology, 1:1 ventriculo-atrial (VA) association and a short RP interval. An electrophysiology study was planned in view of the persistent tachycardia.

**Figure 1:**
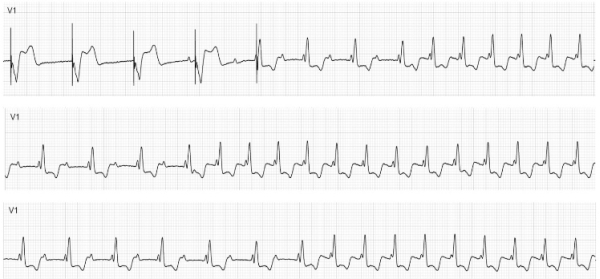
A different instance of spontaneous onset of AVNRT without a premature beat is shown in each panel.

In the electrophysiology lab, he had tachycardia at the beginning of the procedure. The tachycardia could be easily terminated with ventricular overdrive pacing, and on termination of the tachycardia he was found to have sinus rhythm with a PR interval of 380 ms and broad QRS of RBBB morphology. However, tachycardia would always reinitiate spontaneously after a few beats of sinus rhythm (Figure 1). During sinus rhythm, the atrio-his (AH) interval was  274 ms and the his-ventricular (HV) interval was 49 ms. Atrial programmed stimulation could not be performed as any attempt at atrial pacing immediately resulted in tachycardia initiation. Ventricular pacing showed decremental VA conduction with central atrial activation, VA Wenckebach block at 410 ms and VA effective refractory period of 600/360 ms.

**Figure 2:**
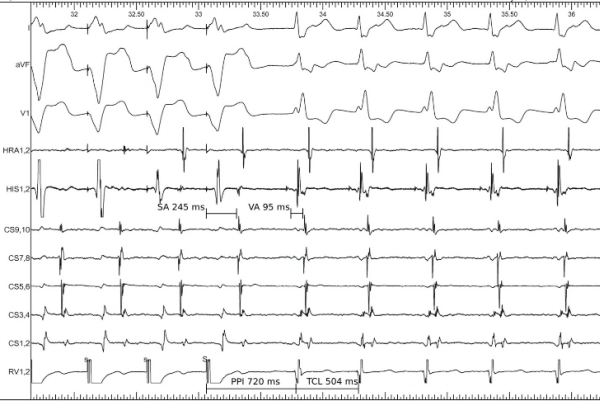
Response of tachycardia to RV overdrive pacing confirming the diagnosis of AVNRT.

Intracardiac electrograms during tachycardia showed 1:1 VA association with VA interval of 95 ms and central atrial activation. RV overdrive pacing at a cycle length (CL) slightly shorter than the tachycardia CL showed a ventricle-atrium-ventricle (VAV) response on termination of pacing with post pacing interval 216 ms longer than the tachycardia cycle length and VA interval was 150 ms longer than stimulus-atrial (SA) interval, all consistent with typical slow-fast atrioventricular nodal reentrant tachycardia (AVNRT) (Figure 2).

Due to the incessant nature of the tachycardia, mapping had to be performed in tachycardia in the region of the slow pathway guided by fluoroscopy and electrograms. Ablation near the os of the coronary sinus was accompained by junctional beats. Post ablation, patient remained in sinus rhythm. AH and HV intervals were 228 ms and 50 ms respectively. Atrial programmed stimulation showed no dual pathway physiology and tachycardia could not be induced with atrial premature stimuli or burst pacing.

## Discussion

AVNRT usually presents as a paroxysmal form of tachycardia with each episode lasting for a short period of time. We could find only one report of incessant presentation of AVNRT [1]. In patients with poor antegrade AV nodal conduction, AVNRT can present as a slow tachycardia [2]. In addition, the poor or absent antegrade fast pathway conduction in our patient caused recurrent spontaneous tachycardia induction without premature stimuli or AH prolongation and resulted in the presentation as an incessant tachycardia. Although the symptoms were mild due to the slow rate, the persistent tachycardia is likely to result in development of ventricular dysfunction over time and therefore required treatment. While it was initially considered that fast pathway ablation should be the treatment in such patients with a prolonged PR interval at baseline, it has been shown that slow pathway ablation is safe and does not result in worsening of antegrade conduction [3]. Preserving antegrade AV nodal conduction was not critical in our patient, however the AH and PR intervals did not change after the successful slow pathway ablation.
